# Orthognathic surgery-related nerve injuries: analysis and treatment modalities

**DOI:** 10.22514/jofph.2026.023

**Published:** 2026-03-12

**Authors:** Yaara Yaniv-Klein, Nadav Grinberg, Amir Shuster, Clariel Ianculovici, Shlomi Kleinman, Reema Mahmoud, Oren Peleg

**Affiliations:** ^1^The Maurice and Gabriela Goldschleger School of Dental Medicine, Gray Faculty of Medical & Health Sciences, Tel Aviv University, 6997801 Tel Aviv, Israel; ^2^Department of Oral & Maxillofacial Surgery, Tel-Aviv Sourasky Medical Center, Gray Faculty of Medical & Health Sciences, Tel Aviv University, 69978 Tel-Aviv, Israel

**Keywords:** Inferior alveolar nerve, Low-level laser therapy, Neurosensory deficit, Orthognathic surgery, Trigeminal nerve injury, Vitamin B supplementation

## Abstract

**Background**: Orthognathic surgery, while highly effective in correcting dentofacial deformities, inherently poses a risk of neurosensory complications. Despite the growing volume of these surgical procedures, real-world data remain limited concerning the precise incidence, clinical course, and efficacy of various therapeutic interventions for nerve injuries in this specific context. This study aims to delineate the incidence, anatomical distribution, and treatment outcomes of trigeminal nerve injuries observed after orthognathic surgery within a tertiary care center. **Methods**: This retrospective cohort study involved 287 patients who underwent orthognathic surgical procedures. Data, including demographic, surgical, and follow-up course, were extracted from electronic medical records. Nerve injuries were specifically identified based on documented postoperative sensory complaints localized to the distribution of the maxillary (CN V2) or mandibular (CN V3) divisions of the trigeminal nerve. Data regarding post-operative treatment using different modalities were collected and analyzed. **Results**: Our retrospective review identified 17 nerve injuries based on documented postoperative sensory complaints within the CN V2 or CN V3 distributions. The various treatment modalities employed included low-level laser therapy (LLLT), corticosteroids, and vitamin B supplementation. Detailed outcomes regarding the incidence and distribution of these injuries were analyzed. **Conclusions**: Post-orthognathic surgery trigeminal neurosensory deficits, while not common, are clinically significant. Our findings suggest that LLLT may offer a therapeutic advantage in managing these deficits, though achieving complete neurosensory recovery remains a challenge.

## 1. Introduction

Orthognathic surgery is frequently performed to address various jaw and facial deformities. These deformities can develop in both childhood and adulthood, negatively impacting the patient’s appearance and function. The procedure is broadly accepted, with a significant rise in its application for correction deformities [1].

As with any surgical intervention, orthognathic procedures carry inherent risks of complications that may manifest during the intraoperative or postoperative periods. A review of existing literature highlights several common adverse events linked to these procedures. These include, but are not limited to, hemorrhage, temporomandibular joint disorders, infections, nerve injuries, complications related to fixation hardware, skeletal relapse, nasal deformities, dental and anesthesiological complications [2, 3]. While these surgeries generally achieve excellent esthetic and functional results, nerve injury remains one of the most clinically significant postoperative complications [4].

Nerve injuries in orthognathic surgery are typically classified as sensory or motor, depending on the function affected. Sensory nerve injuries impair the patient’s ability to detect tactile, thermal, or pain stimuli in the innervated area. These injuries are far more common due to the proximity of major sensory branches—such as the inferior alveolar, mental, and infraorbital nerves—to common osteotomy sites. Motor nerve injuries, by contrast, lead to weakness or paralysis of the affected muscles and, although less frequent, can result in considerable functional and esthetic consequences when branches of the facial nerve are involved [5].

Assessing sensory damage after surgery is often challenging. Subjective patient reports may underestimate the true extent of neurosensory impairment; for instance, while one study found that 46% of patients reported partial recovery within the first year, objective testing revealed more significant residual deficits [6]. The incidence of permanent nerve damage varies with the type of osteotomy and the nerve involved, though most complications are temporary and resolve without intervention [7].

Anatomical complexity and the limited visibility associated with intraoral surgical approaches increase the potential for nerve injury. The nerves most frequently affected are branches of the trigeminal nerve (CN V2 and CN V3), including the infraorbital, buccal, lingual, inferior alveolar, and mental nerves [3, 6, 8, 9]. Of these, the inferior alveolar and lingual nerves are the most commonly injured, with the inferior alveolar nerve particularly vulnerable due to its anatomical position within the mandibular canal [10, 11].

The risk of nerve injury varies by procedure. Le Fort I osteotomy, performed to reposition the maxilla, generally carries a low risk to the infraorbital nerve [12]. Nonetheless, nerve traction or neurovascular fascia shortening may occur from improper soft-tissue dissection or excessive maxillary suspension [13]. In contrast, bilateral sagittal split osteotomy (BSSO), frequently used to correct mandibular deformities, poses a substantially higher risk of inferior alveolar nerve injury [14]. Other mandibular procedures—such as vertical ramus osteotomy, anterior subapical osteotomy, and genioplasty—have also been linked to a notable risk of neurosensory disturbance [15].

The purpose of this study is to delineate the incidence, anatomical distribution, and treatment outcomes of trigeminal nerve injuries observed after orthognathic surgery within a tertiary care center.

## 2. Methods

### 2.1 Study group and data collection

This retrospective study was conducted based on data collected from the medical records of patients who underwent orthognathic surgery in Tel-Aviv Sourasky Medical Center (TASMC), Israel, between January 2010 and December 2024. Inclusion criteria were all patients treated with orthognathic surgery at our department during the study period. Patients under 18 years old and patients lacking judgment were excluded from this study.

As this was a complete census of the source population. Given the retrospective design, primary outcome precision was assessed. Operations were performed by one or two specialized surgeons, together with an oral and maxillofacial resident. Any patient who had undergone orthognathic surgery, LeFort-I osteotomy, sagittal split osteotomy (SSO), intraoral vertical ramus osteotomy (IVRO), or Genioplasty was noted. Collected data include age, sex, duration of surgery, surgical procedures, hospitalization days, intraoperative and postoperative complications with emphasis on nerve injuries, follow-up duration, and further treatments needed. Patients were evaluated daily during their hospital stay. After discharge, the patients were evaluated both clinically and radiographically during follow-up visits. For each group, both dependent and independent variables were collected from the medical files into structured Excel sheets.

### 2.2 Variables and standardization

Exposure/procedures: Type of osteotomy (Le Fort I; BSSO/SSO; IVRO), genioplasty (yes/no; advancement *vs.* reduction/setback), and operative time (minutes), degree of mandibular advancement or setback (mm), occurrence of a bad split, and documentation of inferior alveolar nerve exposure or manipulation. Primary outcome. Postoperative trigeminal neurosensory deficit (yes/no), defined as a documented clinician-verified complaint consistent with CN V2 or CN V3 distribution (*e.g.*, lower lip/chin for inferior alveolar nerve (IAN); upper lip/columella for infraorbital nerve (ION)), recorded during inpatient rounds or outpatient follow-up. When available, objective bedside tests (light touch, two-point discrimination) were extracted; otherwise, standardized subjective reports were used, as per institutional practice. Other variables. Age, sex, length of stay, perioperative dexamethasone (20 mg standard protocol), postoperative adjuvant therapy (LLLT parameters; vitamin B; corticosteroids), and follow-up duration.

Data standardization: All information was obtained from the institutional electronic medical records (EMR), which use standardized templates for surgical and follow-up documentation. Variables were extracted using predefined definitions and reviewed independently by two investigators. Any discrepancies were resolved by consensus. Because all data originated from the same EMR system and surgical department, the reporting format and terminology were consistent across cases.

### 2.3 Statistical analysis

Statistical analysis was performed in SPSS 28.0 (IBM, Armonk, NY, USA). Descriptive statistics reported categorical variables as numbers and percentages. The Shapiro-Wilk test was used to evaluate the normal distribution of continuous variables, which were presented as medians and interquartile ranges (IQRs). The Kruskal-Wallis test and the Mann-Whitney test were used to compare continuous variables between study subgroups. Fisher’s exact test and the Chi-square test were applied to compare categorical variables. All statistical tests were two-sided with a 95% level of significance.

Variable-specific handling: Operative time analyzed as a continuous variable; procedure type as categorical (Le Fort I *vs.* BSSO/SSO *vs.* IVRO; genioplasty yes/no and type); adjuvant therapy categorized (LLLT, vitamin B, corticosteroids, none).

Missing data: Outcome missingness (*e.g.*, lost to follow-up) was treated as missing at random; primary analyses used available cases. A sensitivity analysis excluded patients with incomplete outcome follow-up to assess the robustness of incidence and distribution estimates.

## 3. Results

The study cohort included 287 orthognathic procedures, out of which 56% were female. Of these, 106 procedures involved single-jaw surgeries, and 181 were bimaxillary interventions. The majority of the procedures comprise combination of LeFort-I and IVRO (55.1%), followed by isolated LeFort-I (30%). 39 patients underwent an additional Genioplasty procedure (13.5%), with 33 having advancement genioplasty and six undergoing reduction or setback genioplasty. 13 patients had genioplasty as the only mandibular procedure, combined with maxillary surgery (Fig. 1).

**Fig. 1.** Distribution of the orthogenetic surgeries. IVRO: Intraoral vertical ramus osteotomy; SSO: Bilateral Sagittal Split Osteotomy.

Postoperative neurosensory disturbances were documented in 5.9% of cases (17/287; 95% Confidence interval (CI), 3.7%–9.3%). These cases involved hypoesthesia in the sensory distribution of the maxillary (CN V2) or mandibular (CN V3) branches of the trigeminal nerve. 15 of the 17 (88.2%) cases were mandibular in origin (CN V3), manifesting as hypoesthesia in the lower lip, chin, or cheek. In comparison, two cases (11.8%) involved CN V2 distribution, presenting as numbness of the upper lip and columella (Fig. 2). The affected patients were predominantly female (58.8%), and the median age was 22 years (IQR: 20–29 years). No significant epidemiological predictors of neuropathy were identified.

**Fig. 2.** Neurosensory deficit incidence by procedure performed. IVRO: Intraoral vertical ramus osteotomy; BSSO: Bilateral Sagittal Split Osteotomy; CI: confidence interval.

Of the 17 patients with nerve deficits, 14 (82.2%) received 20 mg of preoperative dexamethasone, administered as a standard anti-inflammatory prophylaxis. Postoperative therapeutic interventions varied: Eight patients (47.06%) received Low-Level Laser Therapy (LLLT) administered using a 970-nm diode laser (Dental diode laser, Sirolaser Blue, Sirona, Charlotte, NC, USA) in continuous mode, 2 W output, 10 seconds per point, Duty cycle 50%–100%, 20 Hz, delivering approximately 10–20 J/cm^2^ per point. Applications were performed extraorally along the mental foramen and mandibular canal projection, with 6–8 sessions over a 3–4-week period as per the institutional protocol. Among these, six patients (75%) reported subjective improvement in sensation; however, complete sensory restoration was not achieved. One patient was treated with additional Vitamin B complex therapy, which consisted of oral supplementation with B1 (thiamine, 100 mg), B2 (Riboflavin, 100 mg), B3 (niacinamide, 100 mg), B6 (pyridoxine, 100 mg), and B12 (cyanocobalamin, 200 μg) once daily for 30 days. In nine cases, no postoperative treatment was administered. Three (33%) of these patients reported subjective improvement in sensation; however, full sensory restoration was not achieved. Three (33%) of these patients experienced persistent sensory deficits, while outcome data were unavailable for the remaining three (33%) due to incomplete follow-up (Fig. 3). In a sensitivity analysis excluding cases with incomplete follow-up, the observed incidence and distribution of neurosensory deficits were materially unchanged.

**Fig. 3.** Neurosensory deficiency treatment modalities and outcomes. LLLT: Low-level laser therapy.

A detailed breakdown of each case, including surgical approach, anatomical site of sensory loss, perioperative management, and sensory recovery trajectory, is presented in Table 1. The incidence of sensory impairment was notably higher in cases involving bilateral sagittal split osteotomy (BSSO) or IVRO combined with LeFort-I osteotomy. In contrast, isolated LeFort-I osteotomies were associated with a lower incidence and milder sensory involvement. Two cases of neurosensory loss were associated with genioplasty.

**Table 1.** Characterization of the cases and the recovery process.

Sex	Age (yr)	Type of surgery		Genioplasty	Site of hypoesthesia	Preoperative steroids	Postoperative treatment	Improvement of sensation
		Maxilla LeFort-I	Mandible IVRO/BSSO					
F	18	LeFort-I	BSSO	Yes	Hypoesthesia in the lower lip on the left side	Dexamethasone (20 mg)	LLLT	Follow-up data unavailable
M	26	LeFort-I	IVRO	No	Hypoesthesia in the lower lip on both sides	Dexamethasone (20 mg)	No treatment	No improvement in sensation
F	34	-	BSSO	No	Hypoesthesia in the lower lip on both sides	-	No treatment	Follow-up data unavailable
F	36	LeFort-I	IVRO	No	Hypoesthesia in the lower lip on the right side	Dexamethasone (20 mg)	No treatment	Lack of information due to unavailability
M	50	LeFort-I	IVRO	No	Hypoesthesia in the lower lip on the left side	Dexamethasone (20 mg)	LLLT	Improvement in sensation without full recovery of sensation compared preoperative
F	17	LeFort-I	IVRO	No	Hypoesthesia in the lower lip on the left side	-	LLLT	Improvement in sensation without full recovery of sensation compared preoperative
M	21	LeFort-I	IVRO	No	Hypoesthesia in the lower lip and cheek on the right side	Dexamethasone (20 mg)	No treatment	No improvement in sensation
F	19	LeFort-I	IVRO	No	Hypoesthesia in the lower lip on the right side	Dexamethasone (20 mg)	LLLT	Improvement in sensation without full recovery of sensation compared preoperative
F	24	LeFort-I	IVRO	Yes	Hypoesthesia in the area of the columella and the upper lip	Dexamethasone (20 mg)	No treatment	Follow-up data unavailable
F	19	LeFort-I	IVRO	No	Hypoesthesia in the lower lip on the right side	Dexamethasone (20 mg)	LLLT	No improvement in sensation
M	25	LeFort-I	IVRO	No	Hypoesthesia in the lower lip on the right side	Dexamethasone (20 mg)	No treatment	No improvement in sensation
M	21	-	BSSO	No	Hypoesthesia in the lower lip on both sides	-	No treatment	Improvement in sensation without full recovery of sensation compared preoperative
F	21	LeFort-I	IVRO	No	Hypoesthesia in the tongue on the right side	Dexamethasone (20 mg)	No treatment	Improvement in sensation without full recovery of sensation compared preoperative
F	29	LeFort-I	IVRO	No	Hypoesthesia in the area of the columella and the upper lip on the right side	Dexamethasone (20 mg)	LLLT + vitamin B	Improvement in sensation without full recovery of sensation compared preoperative
M	20	LeFort-I	IVRO	No	Hypoesthesia in the lower lip on the right side	Dexamethasone (20 mg)	LLLT	Improvement in sensation without full recovery of sensation compared preoperative
M	30	LeFort-I	IVRO	No	Hypoesthesia in the lower lip on the left side	Dexamethasone (20 mg)	No treatment	Improvement in sensation without full recovery of sensation compared preoperative
F	22	LeFort-I	IVRO	No	Hypoesthesia in the lower lip on the left side	Dexamethasone (20 mg)	LLLT	Improvement in sensation without full recovery of sensation compared preoperative

*IVRO: Intraoral vertical ramus osteotomy; BSSO: Bilateral Sagittal Split Osteotomy; LLLT: Low-level laser therapy; M: Male; F: Female.*

The median operative time among patients with nerve injury was longer (230 minutes, IQR: 210–255) compared to the overall cohort (198 minutes, IQR: 180–220), suggesting a potential correlation between surgical duration and neural morbidity, although statistical analysis was not feasible due to the limited sample size of affected cases (Fig. 4).

**Fig. 4.** Operative time comparison between neurosensory injured patients versus the entire cohort.

The mean mandibular movement among patients with postoperative neurosensory deficit was 4.3 ± 1.3 mm, compared with 4.5 ± 1.7 mm in those without a deficit; no significant difference was found. A bad split occurred in one case, and two patients required intraoperative nerve manipulation due to the exposure of the inferior alveolar canal. All three experienced transient sensory disturbances that only partially improved over follow-up. No other intraoperative complications were reported.

## 4. Discussion

The present single-center retrospective analysis provides contemporary data on the incidence, distribution, and early management of trigeminal sensory deficits after orthognathic surgery. Our findings demonstrate a 5.9% incidence of clinically documented hypoesthesia or paresthesia within the maxillary (V2) or mandibular (V3) divisions of the trigeminal nerve. Our findings accord with the broader literature showing that permanent IAN deficits after BSSO are uncommon, whereas temporary postoperative deficits are frequent [5, 14]. In previous retrospective reports of BSSO surgeries, immediate postoperative neurosensory deficit affected 60%–75% of patients, but persistent deficits beyond long-term follow-up were present in 6 to 14% of cases [16, 17, 18]. Systematic reviews similarly conclude that reported prevalence varies with methods and follow-up, but that long-term permanent sensory loss after BSSO is relatively low compared with early postoperative neurosensory deficit [19]. These data justify explicitly stating that, although the overall prevalence of permanent BSSO-related nerve injury is low, temporary neurosensory deficit is common and should be anticipated and counseled preoperatively. The modest prevalence observed in our data may indeed reflect meticulous surgical techniques and our institutional preference for IVRO procedures in Class III corrections, which are consistently associated with fewer IAN injuries than BSSO [15, 20, 21]. However, differences in study design should also be considered when interpreting these findings. Many previous investigations reporting higher incidences were prospective studies specifically designed to evaluate sensory alterations, often employing standardized neurosensory testing and prolonged follow-up protocols. In contrast, the retrospective nature of our analysis, combined with reliance on clinical documentation rather than systematic testing, may have contributed to an underestimation of transient or mild neurosensory changes. Therefore, the apparent discrepancy between our results and those of more targeted sensory studies likely reflects methodological variability rather than solely surgical or institutional factors [22, 23].

Consistent with the pathophysiology of traction, compression, and thermal injury during mandibular osteotomies, most deficits involved the IAN distribution to the lower lip and chin. The single infra-orbital nerve deficit following a LeFort-I osteotomy underscores the comparatively lower risk to V2, attributed to the generous canal diameter and limited manipulation of the infra-orbital bundle during surgical movements [12]. Operative time may represent a predictor of injury. The median operative time for procedures complicated by nerve injury (230 min, 210–255) exceeded that of uneventful cases (198 min, 180–220). Surgical duration has repeatedly emerged as a surrogate marker for technical difficulty and consequent neural traction, with thresholds of 240 min significantly associated with persistent IAN paresthesia [17, 24].

Treatment options for nerve injuries following orthognathic surgery vary depending on the type and severity of the injury. These options include observation and non-invasive therapy for temporary injuries, as well as physiotherapy, local electrical stimulation, low-level laser therapy (LLLT), vitamin B supplementation, and corticosteroids.

LLLT promotes nerve regeneration through photobiomodulation of cellular processes [25]. LLLT appears to improve cellular metabolism and reduce inflammation, all of which support neuronal regeneration and recovery [26]. Studies have explored LLLT’s potential in nerve regeneration and infection prevention after orthognathic surgery, using various laser features like wavelength, energy density, duration, and frequency [27, 28]. LLLT was applied postoperatively to affected nerves. Sensory tests, electromyography, nerve conduction, and patient reports were used to assess outcomes [29]. There are positive outcomes regarding LLLT’s efficacy in nerve regeneration following orthognathic surgery, including improved sensory recovery and accelerated nerve conduction in patients who received LLLT compared to control groups [29]. Studies by Santos *et al*. [29], Sharifi *et al*. [30] evaluated the effectiveness of LLLT on sensorineural recovery after BSSO, showing a general improvement of sensation over time. However, areas receiving LLLT showed greater and accelerated improvement in general sensitivity, pain discrimination, directional discrimination, and two-point discrimination [30]. These findings were supported by further investigations, which concluded that LLLT following orthognathic surgery-related nerve injury may facilitate faster recovery from nerve injuries and improve patient satisfaction, making it a potentially valid treatment option [27, 31].

Vitamin B compounds are an alternative treatment for nerve injuries, serving as coenzymes in nerve metabolism and regeneration. Thiamine (B1) sustains energy production [32], Pyridoxine (B6) functions in neurotransmitter synthesis and balances nerve metabolism, and cobalamin (B12) contributes to DNA synthesis and myelin maintenance. Collectively, these mechanisms contribute to nerve healing and regeneration [33, 34]. Several studies have linked vitamin B supplements after orthognathic surgery to better sensory recovery, less discomfort, and improved nerve conduction [35]. By assessing methylcobalamin, the activated form of Vitamin B12, it was found to promote neurite outgrowth and neuronal survival more than other vitamin B12 analogs. A rat sciatic nerve injury model reveals that methylcobalamin promotes neuronal survival in a concentration-dependent manner, with significant effects observed at concentrations of 100 nM or higher [36].

A randomized controlled trial comparing treatment modalities in 20 patients with paresthesia resulting from inferior alveolar nerve injury following third molar extraction found a significant neurosensory improvement in the LLLT group compared to the vitamin B12 group. The improvement was observed in both subjective (visual analog scale) and objective (light touch and two-point discrimination) neurosensory evaluations [37].

Steroids are known as anti-inflammatory and immunomodulatory agents that reduce edema, potentially creating a more favorable microenvironment for nerve regeneration. Additionally, they may modulate neurotrophic factors and reduce scar tissue formation, thereby facilitating nerve healing [38]. Previous studies have reported good outcomes of steroid therapy for nerve regeneration following orthognathic surgery. Improved sensory recovery, reduced inflammation, and enhanced nerve conduction were observed in patients receiving steroid treatment [39, 40].

In our department, Perioperative care routinely includes a preoperative dexamethasone regimen to mitigate inflammation and edema. Postoperative management was individualized: some patients received LLLT and described symptomatic improvement short of complete recovery, whereas others were managed expectantly. Collectively, these findings suggest a potential adjunctive role for photo-biomodulation in selected neuropraxic presentations.

Despite a thorough search, we found no published, step-by-step protocol dedicated explicitly to managing IAN neurosensory deficits, nor after orthognathic surgeries or third-molar extractions. Existing frameworks either address trigeminal nerve injuries broadly, include early surgical decision points, or offer narrative recommendations without operational details. This evidence gap highlights the need for a prospectively validated, conservative-care pathway. Until then, it supports adapting general trigeminal injury algorithms and establishing service-level standards for documentation, serial neurosensory testing, symptom-guided pharmacotherapy, and time-based reassessment.

Although no significant difference in mandibular movement was observed between patients with and without neurosensory deficits, isolated intraoperative events such as a bad split or nerve manipulation appeared to coincide with transient sensory impairment. These findings are consistent with the mechanisms described by Bertagna *et al*. [17], who identified intraoperative nerve handling and bone segment displacement as key predictors of postoperative neuropathy. Future prospective studies should systematically record and quantify such variables to refine risk modeling and preventive strategies.

Several limitations constrain the present study. First, the retrospective design introduces potential information bias, as shown by incomplete follow-up in four out of nine injured patients. Second, neurosensory assessments mainly depended on subjective patient reports, with objective tools like current perception threshold testing or two-point discrimination not routinely used. Third, the small number of events prevented statistical analysis of risk factors or treatment effectiveness. Finally, the lack of a standardized LLLT or vitamin B regimen limits the ability to generalize the findings.

## 5. Conclusions

In conclusion, trigeminal nerve injury following orthognathic surgery, though uncommon, remains a clinically relevant complication that may affect patient comfort and satisfaction. In this study, the inferior alveolar nerve was most frequently involved, reflecting its anatomical vulnerability during mandibular osteotomies. Adjunctive treatments such as low-level laser therapy, vitamin B supplementation, and corticosteroids demonstrated variable but generally favorable effects on sensory recovery, with low-level laser therapy emerging as the most promising modality. However, complete restoration of sensation was rarely achieved, highlighting the need for early recognition, standardized follow-up, and evidence-based management. Future prospective studies are warranted to refine therapeutic parameters and establish structured protocols aimed at optimizing sensory outcomes and improving patient care after orthognathic surgery.
